# Synthesis of advanced aluminide intermetallic coatings by low-energy Al-ion radiation

**DOI:** 10.1038/srep26535

**Published:** 2016-05-19

**Authors:** Mingli Shen, Yan Gu, Panpan Zhao, Shenglong Zhu, Fuhui Wang

**Affiliations:** 1 Institute of Metal Research, Chinese Academy of Sciences, 62 Wencui Road, 110016 Shenyang, China

## Abstract

Metals that work at high temperatures (for instance, superalloys in gas-turbines) depend on thermally grown oxide (TGO, commonly alumina) to withstand corrosion attack. Nickel Aluminide (NiAl) as one superior alumina TGO former plays an important role in protective coatings for turbine blades in gas-turbine engines used for aircraft propulsion and power generation. Lowering TGO growth rate is essentially favored for offering sustainable protection, especially in thermal barrier coatings (TBC). However, it can only be achieved currently by a strategy of adding the third element (Pt or reactive elements) into NiAl during traditional diffusion- or deposition-based synthesis of the coating. Here we present a highly flexible Al-ion radiation-based synthesis of advanced NiAl coatings, achieving low TGO growth rate without relying on the third element addition. Our results expand the strategy for lowering TGO growth rate and demonstrate potentials for ion radiation in advancing materials synthesis.

Modern advanced gas-turbine engines frequently operate at temperatures above the melting point of the superalloy blades by virtue of the use of thermal barrier coatings (TBC, typically consists of NiAl bond coat and yttria-stabilized zirconia top coat) and internal cooling of the underlying blades^1^,^2^,^3^,^4^,^5^. Adherence of TBC is frequently deteriorated due to overthickening of the thermally grown oxide (TGO, alumina) layer formed on the NiAl bond coat, which would induce ultimate failure of the engine. Therefore, lowering alumina TGO growth rate is essentially favored for sustainably safe service of TBC used in aeroengines^2^,^3^,^6^,^7^,^8^. Nevertheless, this can only be achieved currently by a strategy of adding the third element (Pt or reactive element) into NiAl during traditional diffusion-based synthesis of the coating^2^,^4^. The cost and technology complexity added by the strategy not only reduce the productivity but also limit its use in wider application scenarios; thereby there is a need and challenge to achieve low TGO growth rate without relying on that strategy.

Low-energy (<1 keV) inert-gas ion (Ar^+^, He^+^) radiation has been found to be an effective approach to large-area surface nanostructuring of materials for enhancing surface dynamics^9^,^10^,^11^,^12^,^13^,^14^, which offers an opportunity for altering TGO growth kinetics. Incorporating Al ions as radiation sources, radiation enhanced diffusion of Al into substrate metals would thus lead to concurrent surface alloying and nanostructuring on substrate metals, which fosters a new regime for synthesis of advanced aluminide coatings. This article will demonstrate flexible synthesis of advanced aluminde coatings by low-energy Al-ion radiation, focusing on the unique surface morphology and bulk microstructure formed after the radiation as well as the alumina TGO growth behaviors of the Al-ion radiation-synthesized NiAl coatings.

## Results and Discussion

**Figure 1:**
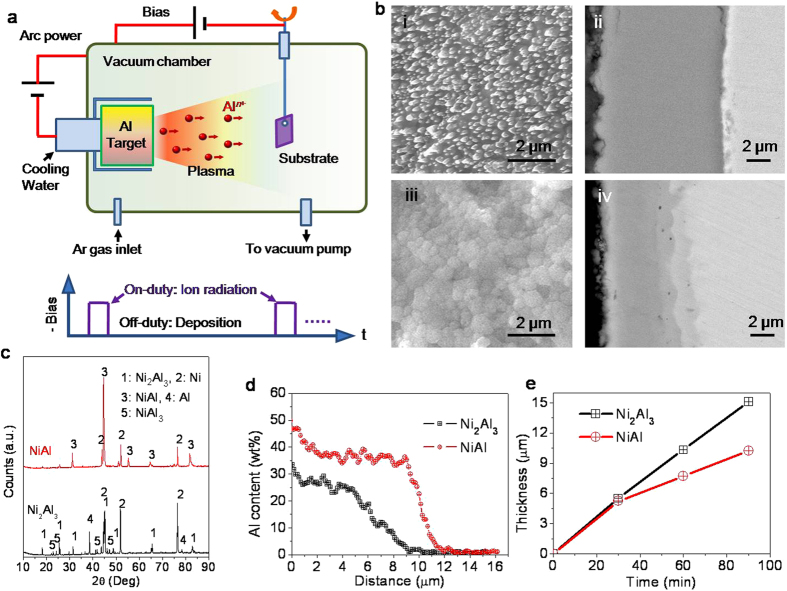
Regime for radiation-based synthesis of aluminide coatings. (**a**) Schematic drawing of the regime. (**b**) SEM images of the surface (i, Ni_2_Al_3_ and iii, NiAl) and cross-section (ii, Ni_2_Al_3_ and iv, NiAl) of aluminide coatings obtained based on this regime. (**c**) XRD patterns of the Ni_2_Al_3_ and NiAl coatings. (**d**) EDS depth profile of Al across the two coatings. (**e**) Growth kinetic curves of the two coatings.

Figure 1a shows a schematic drawing of the regime for the Al-ion radiation-based synthesis of aluminide coatings. High-flux Al-ions are generated by cathodic arc discharge on a pure Al target in vacuum. To enhance the Al-ion radiation, a pulsed accelerating electric field (or pulsed negative bias voltage on substrates, ≤1 kV) is imposed to periodically energize the Al-ions. During on-duty of each pulse Al-ions are energized for radiation, while off-duty corresponds to Al deposition process as illustrated in Fig. 1a. In this regime, substrate metals subject to repetitive alternation between deposition and ion irradiation^1^, which will facilitate rapid aluminide formation as fostered by ion radiation-enhanced diffusion^2^,^3^,^4^ (RED).

To probe nickel aluminide formation process, pure nickel was employed as the substrate at the first step. Evidently, a higher negative bias and duty ratio (DR) correspond to a higher energy level and higher mean flux of energized Al-ions, which both count in aluminide formation. For instance, a ~10 μm thick Ni_2_Al_3_ (Fig. 1bi,ii) layer with ultrafine nodular (~200 nm) surface was obtained after treating for 1 h with arc current of 60 A under a high bias of −1000 V but with low DR of 20% as determined by scanning electron microscopy (SEM, Fig. 1b), X-ray diffraction (XRD, Fig. 1c), and energy dispersive spectroscopy (EDS, Fig. 1d), while a relatively lower bias of −800 V but with much higher DR of 60% produced a ~7 μm thick NiAl (Fig. 1biii,iv) layer with ultrafine-grained (~400 nm) flat surface under the same radiation time and arc current, indicating great flexibilities provided by Al-ion radiation in synthesis of aluminide coatings. The peculiar surface nodule observed on Ni_2_Al_3_ layer stems typically from surface structuring induced by ion beam sputtering (IBS), which pattern would change with ion energy, ion flux etc^5^,^6^,^7^. Moreover, due to insufficient Al-ion radiation, a lower bias or DR could produce a considerable portion of undesired low temperature phases, such as NiAl_3_ or even Al, which were also detected as minor phases on local areas of the Ni_2_Al_3_ layer (Fig. 1c,d). To probe the growth mechanism of the two Ni_2_Al_3_ and NiAl layers, the variation of layer thickness with time is shown in Fig. 1e, which shows unique linear thickening rates. Such growth behavior apparently deviates from those commonly observed parabolic growth rules of aluminide coating obtained by diffusion-based methods^8^,^9^,^10^, indicating a non-diffusion controlled process probably impacted by both IBS and RED. One advantage offered by the linear growth is that it facilitates exact control of coating thickness from the engineering point of view. These results indicate that this regime differs distinctly from either traditional diffusion- or deposition-based process.

**Figure 2:**
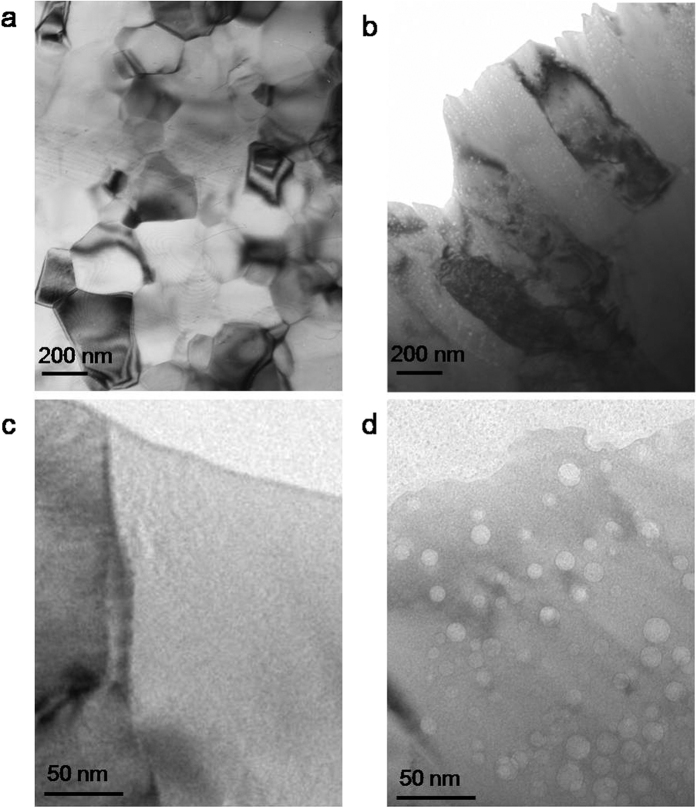
TEM observation of the aluminide coatings. (**a**) Cross-section of the Ni_2_Al_3_ coating, showing ultrafine equiaxed grains. (**b**) Cross-section of the NiAl coating, showing ultrafine columnar grains. (**c**) A magnified image of the Ni_2_Al_3_ coating, showing no nanocavity. (**d**) A magnified image of the NiAl coating, showing abundant randomly distributed nanocavities.

To clarify the characteristic microstructure of the aluminide coatings synthesized based on this regime, we further characterized their cross-sections (Fig. 1bii,iv) by transmission electron microscopy (TEM, Fig. 2). It shows that the Ni_2_Al_3_ layer is composed of ultrafine equiaxed grains of about 200 nm in size which magnitude is in accordance with that of the surface nodules (Fig. 1bi). A more pronounced finding is that the unique NiAl layer is composed of ultrafine columnar grains (200–500 nm) as shown in Fig. 2b. It has been verified that ultrafine, in particular, columnar grains are greatly beneficial for facilitating exclusive alumina TGO formation by providing much more fast diffusion channels at grain boundaries^11^. Although grain-refinement is greatly favored, it is hard to be achieved by those traditional diffusion-based syntheses of the aluminide coatings on commonly coarse-grained metals. However, the radiation-based synthesis paves a way to this object. Moreover, abundant randomly distributed bright spots were observed in the NiAl layer. A magnified TEM image (Fig. 2d) shows that those bright spots are bubble-like nanocavities of 5–20 nm in size. These nanocavities can be seen throughout the NiAl layer but no such nanocavity was found in the δ-Ni_2_Al_3_ layer (Fig. 2c) which was exposed to even higher energetic Al-ion radiation, implicating the necessity of NiAl phase in the formation of nanocavities. One might argue that the nanocavities might be Ar bubbles originated from concurrent Ar ion radiation in a manner similar to He bubbles generation in metals under high level He ion or nuclear irradiation^5^,^12^. Although direct experimental evidences are still missing, we tend to attribute them to nucleation of supersaturated non-equilibrium vacancies induced by ion irradiation other than Ar bubbles. This is because such nanocavities have been observed extensively in bulk NiAl by rapid cooling from high temperatures^13^, implying that in essence NiAl phase is apt to bare these excess lattice defects. Under ion irradiation, injection of vacancies into NiAl lattice would be much easier^2^,^3^,^4^,^5^, thereby bulk nanocavities as vacancy clusters were formed in the NiAl layer even without rapid cooling from high temperatures. These nanocavities can be served as vacancy sources for fostering fast outward diffusion of Al atoms for facilitating the alumina TGO formation.

**Figure 3:**
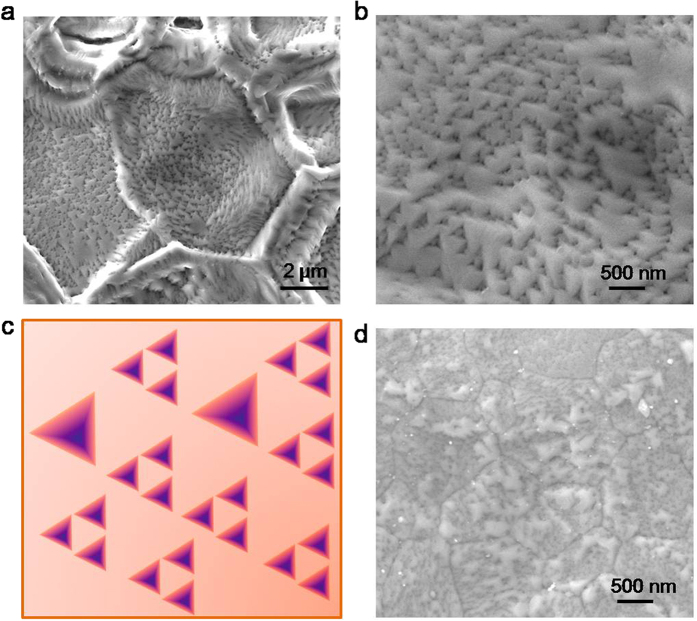
Surface of NiAl coating synthesized by intensified Al-ion radiation. (**a**) SEM image of the surface of NiAl coating synthesized on pure nickel by intensified Al-ion radiation. (**b**) A magnified SEM image of (**a)** shows self-organized surface nanostructuring induced by IBS. (**c**) Schematic illustration of the Sierpinski-triangle like surface pattern. (**d**) SEM image of the surface of NiAl coating synthesized on a commercial Ni-based superalloy K438G by intensified Al-ion radiation, exhibiting similar surface patterning.

To intensify Al-ion radiation, we upgraded the overall Al-ion density by doubling the arc current which in turn favors for a faster synthesis of thick NiAl coatings. Figure 3a,b shows the surface of NiAl coating synthesized for 1 h on pure nickel with arc current of 120 A under bias of −800 V with DR of 60%. Raised grain boundaries can be clearly identified from the surface image, implicating strong IBS and grain coarsening induced by the intensified Al-ion radiation. More notable characteristics featured by the intensified Al-ion radiation is surface nanostructuring in the form of regular inverted triangular pyramid (ITP) pits with size range of 30–150 nm (Fig. 3a), which is distinctly different from the nodular surface as shown in Fig. 1bi. A magnified image shows that not only the shape of each pit but also their arrangement is well-ordered (Fig. 3b). In particular, a relatively larger ITP pit (>100 nm) is actually an assembly of three well-ordered smaller individual pyramid pits (30–60 nm) as schematically illustrated in Fig. 3c, similar to a fractal pattern of the well-known Sierpinski triangle^14^. Meanwhile, less bulk nanocavities were found in the NiAl coating synthesized by the intensified Al-ion radiation, which might be closely related to the surface patterning that confined part of the defects on the surface rather than in the NiAl lattice^6^,^15^. The formation of surface nanoscale ITP pits which bare a great deal of atomic surface steps at their edges is also favored according to recent reports that atomic surface steps are found to be beneficial to suppress alumina TGO growth rate on NiAl^16^. Similar surface patterning was also observed on the surface of a thick (~30 μm) NiAl coating (Fig. 3d) synthesized on a commercial nickel-based superalloy K438G after the intensified Al-ion radiation for 1.5 h under the same condition as mentioned above.

**Figure 4:**
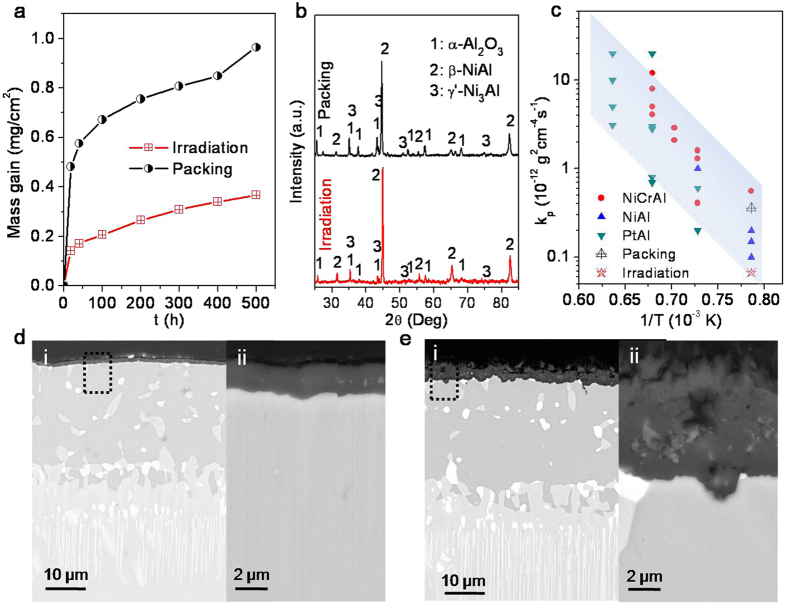
High temperature oxidation of the NiAl coatings. (**a**) Oxidation (1000 °C in air) kinetic curves of samples with two different NiAl coatings synthesized by radiation and traditional packing methods. (**b**) XRD patterns of the two NiAl coatings after oxidation for 500 h at 1000 °C. (**c**) Arrhenius plot of the parabolic constants *k*_p_ obtained from this study and literature, showing low alumina TGO growth rate of the radiation-synthesized NiAl coating. (**d**) SEM image (i, ii) of the cross section of radiation-synthesized NiAl coating after oxidation for 500 h at 1000 °C, showing a thin and dense alumina layer (ii). (**e**) SEM image (iii, iv) of the cross section of traditional packing-synthesized NiAl coating after oxidation for 500 h at 1000 °C, showing a much thicker and porous alumina layer (iv).

To evaluate the alumina TGO growth behaviors, we conducted oxidation tests (1000 °C in air) on the thick NiAl coating synthsized on the superalloy K438G by the intensified Al-ion radiation. For comparison, the tests were also conducted on a NiAl coating synthesized by traditional powder packing method. Figure 4a shows the oxidation kinetic curves measured by mass gain of the samples with the two different types of NiAl coatings. As expected, both of them followed parabolic mass gaining rules throughout the oxidation test for 500 h due to formation of α-Al_2_O_3_ scales as determined by XRD (Fig. 4b). However, the kinetic curve for the sample with radiation-synthesized coating is substantially depressed as compared with that of the traditional packing-synthesized one, indicating a much lower growth rate of the alumina TGO layer grown on the radiation-synthesized NiAl coating. Their parabolic constants *k*_p_, as expressed by: *k*_p_ = (Δ*w*)^2^/*t*, where Δ*w* is specific mass gain and *t* is oxidation time^9^, are shown in an Arrhenius plot (Fig. 4c) together with reported values of those alumina TGO formers^8^,^17^. The value for the radiation-synthesized coating locates at the lower limit side close to that of superior PtAl coatings. It is reported that Pt facilitates outward diffusion of Al in aluminide phase and Pt doping in alumina scale lowers its growth^8^,^9^,^18^,^19^. In this study, we found that such benefits offered by Pt or reactive elements might be also provided by the bulk nanocavities and surface steps that functioned in forming a pure slowly growing α-Al_2_O_3_ scale. Cross-sectional observations on the samples after oxidation for 500 h further verified that thin (~1.8 μm) and dense α-Al_2_O_3_ scale was formed on the radiation-synthesized coating (Fig. 4d) while the packing one (Fig. 4e) grew a much thicker (~5 μm) and porous α-Al_2_O_3_ scale^20^,^21^, which consists well with their mass gains as shown in Fig. 4a.

According to vacancy diffusion mechanism, outward diffusion of Al atoms requires injecting equivalent amount of vacancies from the surface. If sufficient amount vacancies already exist inside NiAl, the outward diffusion of Al atoms would be much easier and faster. Combined with surface nanostructuring and bulk nanocavities, a pure alumina TGO would be established rapidly at the initial oxidation stage and grow slowly latterly in the steady oxidation stage^22^. Our results demonstrate that a structural optimization of NiAl coating offered by ion radiation also functions in lowering alumina TGO growth rate. It also shows that ion radiation not only expands the strategy for lowering alumina TGO growth rate but also offers great flexibilities allowed for synthesis of advanced surface coatings in addition to NiAl.

## Methods

Pure Al (99.99 wt%, weight percent) was used as cathodic target (Cylinder shape with dimension of 100 mm in diameter and 40 mm in height) for generating high flux Al-ions in vacuum by arc discharge (DC current supplier, 10–200 A). Prior to igniting the arc discharge, the vacuum chamber was evacuated to 6 × 10^−3^ Pa pumped by an oil diffusion pump backed by a rotary pump. Substrate samples were suspended and hung in a rotating stand (10 r/min) in front of the target for a mean distance of about 150 mm as shown in Fig. 1a. To energize Al-ions for enhancing ion radiation, pulsed negative bias (1–1000 V, 40 kHz, duty ratio 10–90%) was applied to substrate as shown in Fig. 1a. High purity Ar gas was flowed into the vacuum chamber to maintain the vacuum pressure to the set value of 8 Pa during synthesis of aluminide coatings.

Pure nickel (99.9 wt% in purity) with dimensions of 10 × 15 × 2 mm was used as substrate for probing the of aluminide formation behavior. Prior to synthesis, nickel samples were ground on 1000# SiC abrasive paper followed by ultrasonically rinsing in ethanol for 5 min to eliminate contaminates. Substrate temperature (T_s_, °C) rise was inevitably occurred during ion radiation, which raised approximately linearly with the product of bias (E_b_, V) and duty ratio (η) as described by T_s_ = 0.58 × E_b_ × η + 524 (arc discharge current 60 A), according to measured results by type K thermocouples. For a doubled arc discharge current of 120 A, there was a further rise of substrate temperature of about 120 °C based on the above calculation. To compare the oxidation behavior of the radiation- and traditional powder packing-synthesized aluminide coatings, commercial Ni-based superalloy K438G (weight percent of Ta 1.75, Ti 3.81, Mo 1.77, Cr 16.34, Co 8.38, Al 4.00, C 0.16, W 2.66, Nb 0.76, B 0.01 and Ni in balance) with the same dimensions was used as substrate. The powder packing aluminide coatings were obtained by packing the K438G samples into mixed powders of Fe-Al (98 wt%) and NH_4_Cl (2 wt%) followed by heat treatment at 1000 °C for 2 h.

Phase identification was conducted by XRD (X’ Pert PRO, PANalytical Co., Almelo, the Netherlands, Cu Kα radiation at 40 kV). SEM (InspectF 50, FEI Co., Hillsboro, OR), TEM (JEOL JEM 2010F) and EDS (INCA, X-Max, Oxford instruments Co., Oxford, U.K.) were used for analysis of surface morphology, cross-section and composition of the coatings. Isothermal oxidation tests were conducted at 1000 °C in air in a muffle furnace. Each sample was weighed during oxidation by a precision electronic balance (0.01 mg precision, BP211D, Sartorius, Germany).

## Additional Information

**How to cite this article**: Shen, M. *et al.* Synthesis of advanced aluminide intermetallic coatings by low-energy Al-ion radiation. *Sci. Rep.* **6**, 26535; doi: 10.1038/srep26535 (2016).
